# External treatment of traditional Chinese medicine for myasthenia gravis: A protocol for systematic review and meta-analysis

**DOI:** 10.1097/MD.0000000000025475

**Published:** 2021-04-16

**Authors:** Yuxuan Peng, Bibo Lu, Lu Li, Yuting Pan, Qing Ye, Min He, Weiyin Chen, Xueping Yang

**Affiliations:** a Chengdu Eighth People's Hospital (Geriatric Hospital of Chengdu Medical College); b Hospital of Chengdu University of Traditional Chinese Medicine, Chengdu, China.

**Keywords:** external treatment, meta-analyses, myasthenia gravis, protocols, traditional Chinese medicine

## Abstract

**Background:**

Myasthenia gravis (MG) is an archetypal autoimmune disorder. The conventional treatments for this disease are drugs, plasma exchange, surgical, and so on. However, this disease is difficult to cure. A mass of studies revealed that the external treatment of traditional Chinese medicine (TCM) for MG is a safe and economical approach. The present study conducted a meta-analysis to compare TCM external treatment combined with modern medicine with modern medicine for MG, in order to determine which TCM external treatment intervention has the best relative efficacy, safety, and provide the best evidence for clinical practice.

**Methods:**

PubMed, Cochrane Library, EMBASE, Web of Science, Springer, China National Knowledge Infrastructure (CNKI), Wan-fang database, VIP Chinese Science and Technique Journals Database, the Chinese Bio Medical Database (CBM), and Baidu Scholar were searched. The time of publication was limited from inception to February 28, 2021. Two reviewers independently searched for the selected articles and extract the data. The RevMan V.5.3 statistical software (Cochrane Collaboration) and Stata V.16.0 software were used to conduct the meta-analysis.

**Results:**

The results of the systematic review and meta-analysis will be published in a peer-reviewed journal.

**Conclusion:**

The present study provides a protocol that can be used in the systematic review and meta-analysis, with the intent to inform professionals on the external treatment of TCM for MG. These would lead to investigations on the use of the most external treatment of TCM for MG.

**Trial registration number:**

INPLASY202110083

## 1 Introduction

Myasthenia gravis (MG) is an archetypal autoimmune disorder, which is featured with voluntary muscle weakness and fatigue. This is a rare disorder, but forms the most frequent type of neuromuscular junction disorder.^[1,2]^ The cause of MG remains complicated and unknown. However, in a number of studies, this appears to be correlated to thymus disease. The principal manifestation of MG is blepharoptosis, diplopia, dysphagia, limbs weakness, breathing difficulties, and so on. The characteristics of its symptoms are fluctuations for the whole day, such as feeling lighter after rest or in morning, and feeling worse after activities or at night. Meanwhile, the laboratory test for acetylcholine receptor antibody (AchRAb),^[3]^ interleukin-6, IFN-γ and its mRNA, together with the clinical symptoms, confirms these. In general, for the treatment of this disease, drugs (glucocorticoids, immunosuppressants, cholinesterase inhibitors, intravenous gamma globulin, and so on), plasma exchange and surgery have enabled patients with MG to extend their life. Nonetheless, this disease cannot be cured. Hence, the investigators attempted to identify more treatment methods, and the external treatment of traditional Chinese medicine (TCM) was identified as a more safe and economical approach.

In general, the symptoms of some MG patients continued to repeatedly fluctuate during drug treatment, and this can be delayed for several years. Hormones and other immunosuppressants are needed for its long-term maintenance. The immunosuppressive therapy generally has disadvantages, such as large side effects. Therefore, external therapy is increasingly required to treat MG, such as acupuncture, warm acupuncture, Tuina, point injection, moxibustion, etc. A number of clinical studies have revealed that the combination of Western medicine therapy with TCM external treatment can reduce the recurrence rate of diseases, reduce the occurrence of adverse reactions and complications, and regulate immunity.^[4–8]^

However, these trials were mostly small, sample randomized controlled trials, the trials had great differences between the results, and the experimental conclusions were controversial. Therefore, in order to evaluate the efficacy of integrated TCM external therapy and modern medicine in the treatment of MG, the randomized treatment of MG based on modern medicine combined with TCM external therapy was conducted. Then, a systematic review was performed on the controlled trials, to provide reliable evidence-based medicine for clinical practice.

## 2 Methods and analysis

### 2.1 Protocol registration

The protocol was based on the Preferred Reporting Items for Systematic Reviews and Meta-Analyses Protocols (PRISMA-P) statement guidelines.^[9]^ The present systematic review protocol is registered on the INPLASY website, with registration number: INPLASY202110083. The duration of the study will be adjusted when any relevant information is updated during the study period. Ethical approval was not required.

### 2.2 Types of studies

Merely randomized controlled trials (RCTs) that investigated the effect and (or) safety of the external treatment of TCM on MG in adults were included. Animal mechanism studies, case reports, self-pre-control and post-control, and non-RCTs were excluded.

### 2.3 Types of participants

Patients with MG were included according to any recognized diagnostic criteria. These patients were approximately 18 years old, and the selection was not restricted for gender, ethnicity, race, or disease stage. Patients with the following were excluded: severe cardiovascular diseases, severe liver injury, severe kidney injury, mental illnesses, pregnant or lactating women, etc.

### 2.4 Types of interventions

One or more external treatments of TCM (such as acupuncture, electroacupuncture, transcutaneous electrical nerve stimulation, acupoint catgut embedding, moxibustion, warm acupuncture, acupressure, cupping jar, fire needle, auricular acupuncture, scalp needle, abdominal acupuncture, superficial acupuncture bleeding, acupoint injection, needle knife, point application, Tuina, Traditional Chinese medicine fumigation and washing, and so on) were included, with or without any Western medicine, for the treatment of MG.

For the control group, one or more modern medicines (such as acetylcholine preparation, hormone, immunosuppressant, intravenous immunoglobulin, etc.), placebo control, or no therapy was used. There were no limitations on the intervention approach.

### 2.5 Types of outcomes

#### 2.5.1 The primary outcomes are listed below

1. Effective rate;
2. The Quantitative Myasthenia Gravis (QMG) scores;
3. Adverse events;
4. Quality of life.

#### 2.5.2 The secondary outcomes are, as follows

1. The concentration of acetylcholine receptor antibody (AchRAb) in serum;
2. The change in related immune cells in the serum Traditional Chinese Medicine Syndrome Score Scale (TCMSSS);
3. The serum interleukin-6 level;
4. The level of IFN-γ and its mRNA;
5. Clinical score calculated based on The Chinese Expert Consensus on The Diagnosis and Treatment of Myasthenia Gravis (The clinical score contains the Clinical Absolute Score and Clinical Relative Score).

### 2.6 Data sources and search strategies

A systematic literature search of articles published up to February 28, 2021 was conducted with the assistance of an experienced librarian in the following electronic databases: PubMed, Cochrane Library, EMBASE, Web of Science, Springer, China National Knowledge Infrastructure (CNKI), Wan-fang database, VIP Chinese Science and Technique Journals Database, the Chinese Bio Medical Database (CBM), and Baidu Scholar. All RCTs on the external treatment of TCM, with or without any Western medicine, for the treatment of MG were collected. Merely RTCs in the English and Chinese language were selected for the present study. Unpublished studies were excluded (Table 1).

**Table 1:** Search strategy in Medline.

1.	randomized controlled trial.pt.
2.	controlled clinical trial.pt.
3.	randomized.ab.
4.	placebo.ab.
5.	clinical trials as topic.sh.
6.	randomly.ab.
7.	trial.ti.
8.	1 or 2 or 3 or 4 or 5 or 6 or 7
9.	exp animals/ not humans.sh.
10.	8 not 9
11.	myastheni$.tw.
12.	exp myasthenia gravis/
13.	Myasthenic Syndromes, Congenital/
14.	exp Weizheng/
15.	or/11-14
16.	exp acupuncture therapy/or exp acupuncture/or exp acupuncture points/or exp acupuncture, ear/or exp electroacupuncture/or exp meridians/or exp acupuncture points/or exp moxibustion/
17.	exp Acupuncture/
18.	(acupuncture or acupressure or electroacupuncture).tw.
19.	(meridian$ or acupoint$).tw.
20.	needling.tw.
21.	moxi$.tw.
22.	(moxabustion or moxa or artemisia or mugwort$).tw.
23.	shu.tw.
24.	(shiatsu or tuina or mess$).tw.
25.	(trigger adj3 point$).tw.
26.	or/16–25
27.	complementary medicine.mp.
28.	Alternative Medicine/
29.	alternative medicine.mp.
30.	herbal medicine.mp.
31.	Medicine, Herbal/
32.	Chinese adj5 herbal.mp.
33.	Medicine, Chinese Traditional/
34.	Drugs, Chinese Herbal/
35.	TCM/
36.	Medicine, Oriental Traditional/
37.	or/27–36
38.	external therapy/
39.	external treatment/
40.	external treatment of TCM/
41.	or/38-40
42.	exp catgut implantation at point/
43.	exp acupoint thread implantation/
44.	embed$.tw.
45.	or/42–44
46.	phlebotomy/or bloodletting/
47.	(phlebotomy or bloodletting or blood-letting).tw.
48.	or/46–47
49.	exp fumigation-washing/
50.	(fumi$ or steam$)
51.	or/49–50
52.	cupping therapy.tw.
53.	temperature the p$.tw.
54.	or/52–53
55.	26 or 37 or 41 or 45 or 48 or 51 or 54
56.	10 and 15 and 55

### 2.7 Data extraction

#### 2.7.1 Selection of studies

Two reviewers independently browsed the selected articles, and extracted the following information on Microsoft Excel:

1. essential information: the first author, year of publication, and country;
2. study design: setting, inclusion and exclusion criteria, randomization method, blinding, sample size, and dropouts;
3. participants: gender, age, and disease duration;
4. methodological characteristics: title study design, MG severity, and diagnostic criteria;
5. details of intervention: type of intervention (such as acupuncture, electroacupuncture, transcutaneous electrical nerve stimulation, acupoint catgut embedding, moxibustion, warm acupuncture, acupressure, cupping jar, fire needle, auricular acupuncture, scalp needle, abdominal acupuncture, superficial acupuncture bleeding, acupoint injection, etc.), duration of treatment, and follow-up time;
6. results for the evaluation method.

#### 2.7.2 Dealing with missing data

For missing or insufficient data, the authors were contacted to request for adequate data by email or telephone. If the missing data could not be obtained, trails with missing data were excluded from the data synthesis. Then, a sensitivity analysis was performed to determine the impact of the missing data.

### 2.8 Risk of bias assessment and quality of selected studies

The methodological quality of randomized controlled trials was assessed using the Cochrane Collaboration's risk of bias tool. This tool focused on the following domains: generation of random sequence, allocation concealment, blinding of participants and personnel, incomplete outcome data, duration of follow-up, selective reporting, and other bias. Any disagreements between the reviewers were resolved through discussion, or by seeking advice from a third reviewer. The quality of the evidence was evaluated using the Grading of Recommendations Assessment, Development and Evaluation (GRADE), which determines the quality of the evidence according to the primary outcomes, consistency of the results, imprecision, and publication bias. The strength of the evidence was graded as high, medium, low, and very low quality.

### 2.9 Statistical analysis

When a meta-analysis was possible, the data synthesis was presented using the RevMan V.5.3 statistical software. The statistically data was meaningful when *P* < .05. The SMD with 95% CI was used to evaluate the continuous outcomes, and the RR with 95% CI was used to evaluate the dichotomous data. The fixed effects model (I^2^ < 50%) was used to estimate the RR and MD. The random effects model (I^2^ > 50%) was considered for the indication of substantial statistical heterogeneity, and used for the synthesis of the data, and the subgroup analysis or sensitivity analysis. The funnel plots were indicated for obvious publication bias.

### 2.10 Sensitivity analysis

The sensitivity analysis was conducted to evaluate the robustness and reliability of the pooled results. If adequate data was available for analysis, a sensitivity analysis for the primary outcomes was conducted to test the strength of the review conclusions, which included the quality of the methods and studies, and the impact of the sample size and missing data.

### 2.11 Publication bias

If more than 10 studies were included, the funnel plot was used to determine the publication bias. Egger's test and Begg's test were performed to quantitatively assess the publication bias using the Stata V.16.0 software. The results were estimated based on the Cochrane Handbook for Systematic Reviews of Interventions.

### 2.12 Confidence in cumulative evidence

The strength of the evidence was evaluated based on the Grading of Recommendations assessment, Development, and Evaluation system. The evidence was classified into four levels: high, moderate, low, or very low.

### 2.13 Selection of studies

**Figure 1:**
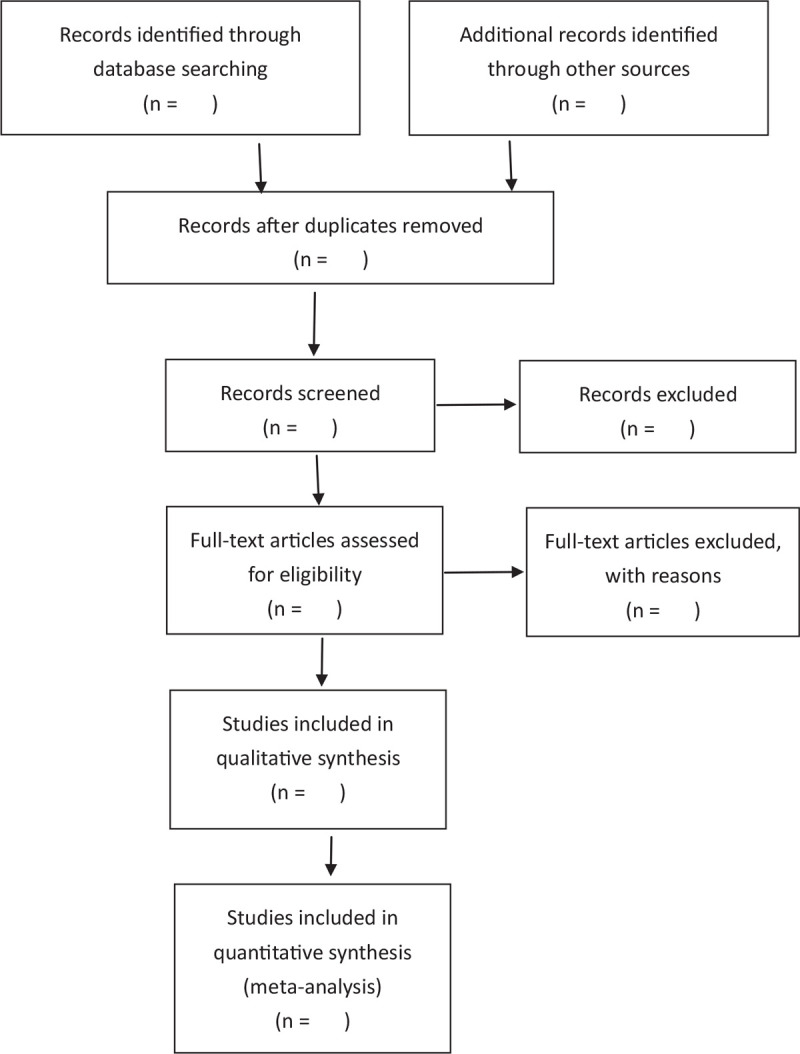
Flow diagram of study selection process.

Two review authors, who were trained in systematic review techniques, independently searched and assessed the titles and abstracts of the potential studies identified through the search strategy for eligibility. If the eligibility of a study was inexplicit from the title and abstract, the article was fully browsed. Studies that did not meet the inclusion criteria for the present review were excluded, and the reasons for the exclusion of studies were recorded. If there was any disagreement, this was resolved through the consensus of all authors. The Preferred Reporting Items for Systematic Reviews and Meta-Analyses (PRISMA) study flowchart was used to record the screening process (Fig. 1).

## 3 Discussion

In recent years, increasing clinical reports have revealed that various external treatments of TCM combined with drugs can achieve certain efficacy in the treatment of MG. However, there is no comparison on the external treatment of TCM for the direct or indirect treatment of MG. Therefore, the present study conducted a meta-analysis to directly or indirectly compare TCM external treatment combined with modern medicine to modern medicine for MG, determine which TCM external treatment intervention has the best relative efficacy and safety, and provide the best evidence for clinical practice.

## Author contributions

**Conceptualization:** Min He.

**Data curation:** Yuting Pan, Qing Ye.

**Formal analysis:** Yuting Pan, Qing Ye.

**Funding acquisition:** Weiyin Chen.

**Investigation:** Lu Li.

**Methodology:** Yuxuan Peng, Bibo Lu.

**Project administration:** Yuxuan Peng, Bibo Lu.

**Resources:** Min He, Xueping Yang.

**Software:** Yuting Pan, Qing Ye.

**Supervision:** Lu Li.

**Validation:** Weiyin Chen.

**Visualization:** Min He, Xueping Yang.

**Writing – original draft:** Yuxuan Peng, Bibo Lu.

**Writing – review & editing:** Lu Li.
